# A case report of a perigraft inflammatory reaction to a Viabahn stent-graft: diagnosis with MRI and treatment with steroids

**DOI:** 10.1186/s42155-020-00140-3

**Published:** 2020-09-20

**Authors:** Tetsuya Yamamoto, Kenzo Uzu, Takahiro Sawada, Tomofumi Takaya, Hiroya Kawai

**Affiliations:** Division of Cardiovascular Medicine, Department of Internal Medicine, Hyogo Prefectural Himeji Cardiovascular Center, 520, Saisho-Kou, Himeji, Hyogo 670-0981 Japan

**Keywords:** Viabahn, Perigraft inflammatory reaction, Magnetic resonance imaging, Steroid, Case report

## Abstract

**Background:**

Perigraft inflammatory reactions to prosthetic graft materials in vascular surgery have been reported; however, to our knowledge, this is the first report of a perigraft inflammatory reaction to a Viabahn stent-graft used in a superficial femoral artery occlusion lesion.

**Case presentation:**

A 76-year-old man with right leg claudication was diagnosed with a right superficial femoral artery occlusion via contrast-enhanced computed tomography. Endovascular treatment included intravascular ultrasound for passing through the true lumen. A 25-cm Viabahn stent-graft (diameter 5 mm) was implanted. The patient developed pain and local swelling of the right thigh 5 days after endovascular treatment. Blood analysis revealed elevated inflammatory marker levels. Magnetic resonance imaging revealed extensive soft-tissue edema and a high perivascular T2 signal around the right superficial femoral artery. Clinical symptoms resolved within 7 days after initiating steroid therapy, which was gradually decreased and halted after 3 weeks. Follow-up magnetic resonance imaging demonstrated substantially reduced inflammation over the following months.

**Conclusions:**

Perigraft inflammatory reaction to a Viabahn stent-graft implant can be immediately diagnosed via magnetic resonance imaging and treated with steroids to reduce the possibility of stent-graft occlusion.

## Background

Perigraft inflammatory reactions to prosthetic graft materials in vascular surgery have been reported (Henry et al. 1994); however, to our knowledge, this is the first report of a perigraft inflammatory reaction to a Viabahn stent-graft (W.L. Gore & Associates, Flagstaff, AZ, USA) used in a superficial femoral artery (SFA) occlusion lesion. Additionally, in the case we present here, the inflammation was diagnosed and monitored using magnetic resonance imaging (MRI) and was effectively treated with steroids.

## Case presentation

**Fig. 1:**
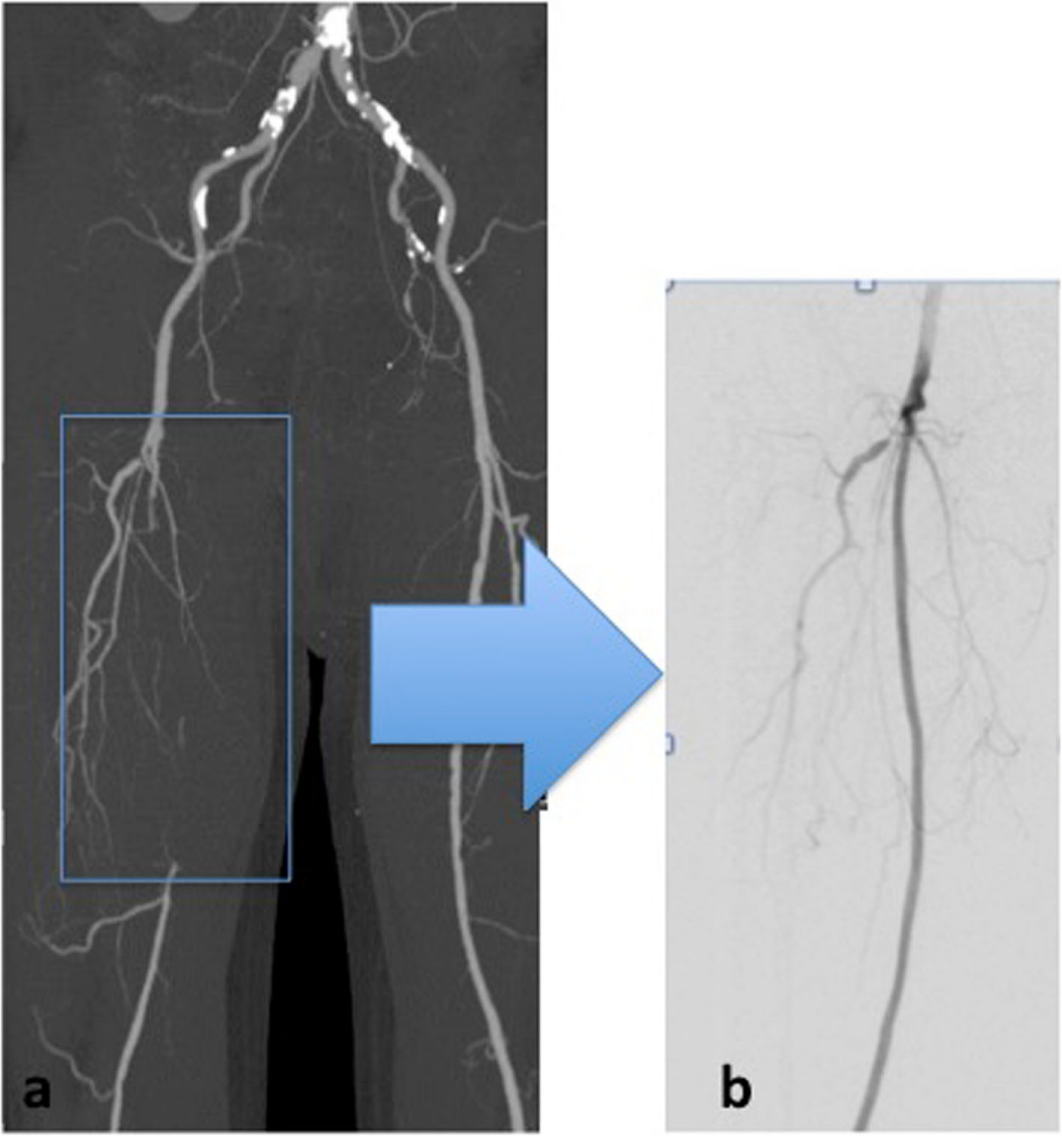
Imaging of the superficial femoral artery occlusion lesion before and after endovascular therapy. **a** Preoperative computed tomography angiography revealing the superficial femoral artery occlusion lesion. **b** Post-stenting angiography revealing no dissection

**Fig. 2:**
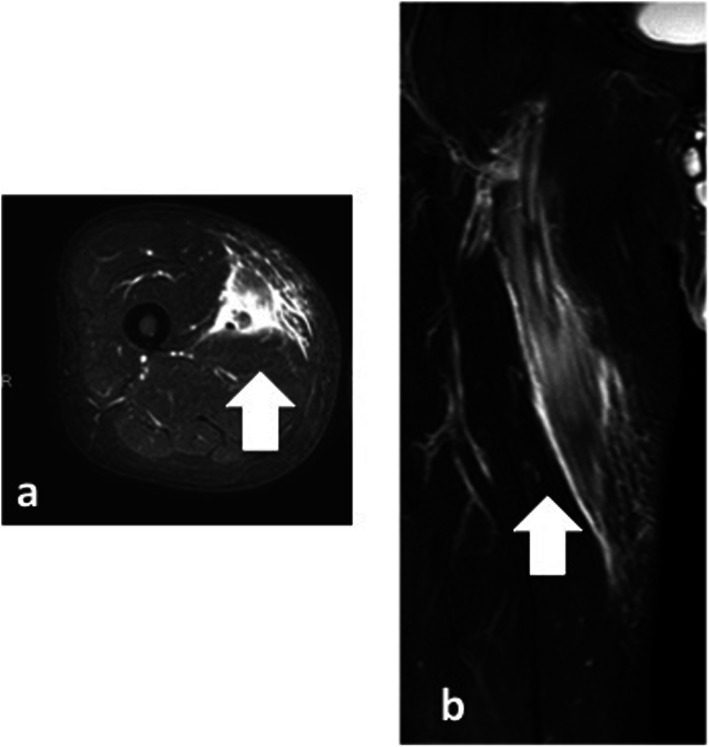
T2-weighted magnetic resonance images of the patient’s thigh. T2-weighted images (**a**, axial; **b**, coronal) revealed perivascular inflammation (arrows: edema of soft tissue) surrounding the superficial femoral artery

**Fig. 3:**
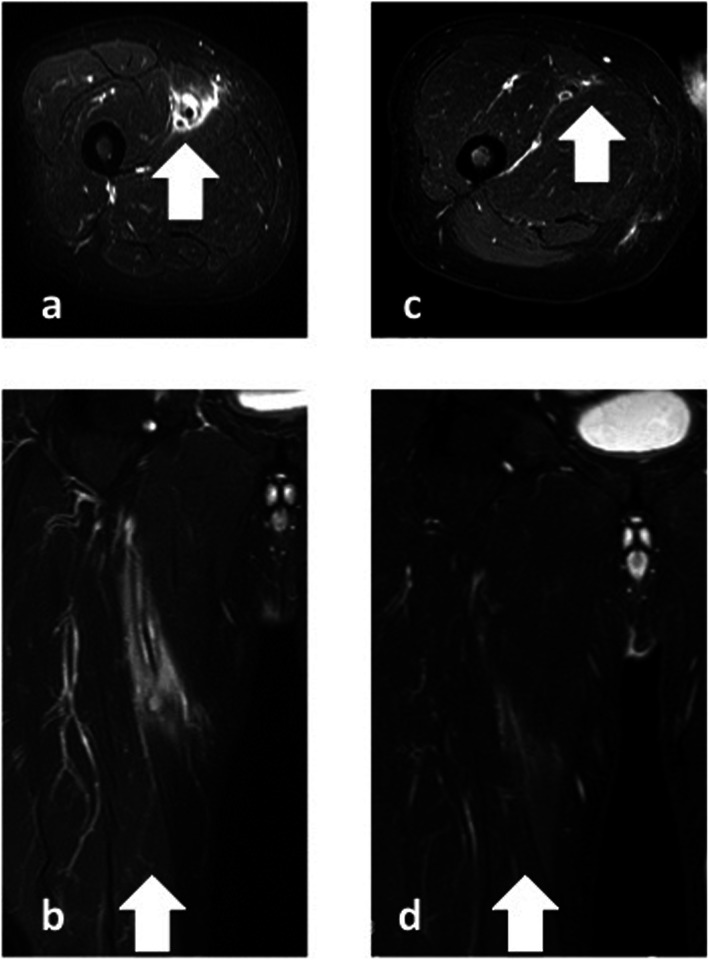
Serial magnetic resonance imaging scans of the right thigh. **a**, **b** Seven days after anti-inflammatory medication was begun. **c**, **d** Three months later. Note the progressive reduction in soft-tissue edema (arrows) surrounding the right superficial femoral artery

A 76-year-old man presented with a peripheral occlusive disease classified as Fontaine Stage IIB. He had a history of hypertension and dyslipidemia, and was a smoker. Preoperative contrast computed-enhanced tomography (CT) revealed a right SFA occlusion (Fig. 1a). We initiated antiplatelet therapy (100 mg/day aspirin and 75 mg/day clopidogrel). Angiography revealed a 16-cm chronic total occlusion of the right SFA. Endovascular treatment (EVT) was performed on the right SFA following intra-arterial injection of heparin (5000 units). A 6-French (Fr) guiding sheath was inserted via the left femoral artery. Intravascular ultrasound (IVUS) was used for passing through the true lumen. Thereafter, a 4-mm semi-compliant balloon was used for pre-dilatation and IVUS revealed no major dissections. A 25-cm Viabahn (diameter 5 mm) was implanted and a 5-mm non-compliant balloon was used for post-dilatation. On the final angiogram, no edge dissection was visible, and good flow was confirmed (Fig. 1b). The patient’s symptoms improved, and he was discharged after 4 days; however, he developed pain and local swelling of the right thigh 6 days after EVT. Venous thrombosis was excluded by ultrasound imaging. He had no fever, negative blood culture findings and negative procalcitonin levels (0.1 ng/ml; standard value is ≤0.5 ng/ml). However, blood analysis revealed elevated inflammatory marker levels: white blood cell (WBC) count was 12,000 cells/μl (standard values are 4500–7500 cells/μl) and C-reactive protein levels were 11.83 mg/dl (standard value is ≤1.0 mg/dl). MRI revealed extensive soft-tissue edema and a high perivascular T2 signal around the right SFA (Fig. 2); however, the stent-graft remained patent. This indicated possible perigraft inflammation. The clinical symptoms were severe and there was a risk of occlusion of the stent-graft. We initiated steroid therapy (prednisolone, 20 mg/day) 2 days after the symptoms started and decreased it as soon as possible. Clinical symptoms resolved within 7 days after initiating steroid therapy, and follow-up MRI revealed regression of soft-tissue edema (Fig. 3a, b). Therefore, the prednisolone was decreased to 10 mg/day and tapered by 5 mg per week until it was halted. Follow-up MRI demonstrated marked resolution of inflammation over 3 months (Fig. 3c, d).

## Discussion and conclusions

It is well known that local and systemic inflammation may occur after stent-graft implantation; however, to our knowledge, this is the first description of an inflammatory reaction to a Viabahn stent-graft.

There are several possible explanations for the observed inflammation. One possibility is bacterial contamination of the stent-graft during the intervention. It is difficult to differentiate between infection and a perigraft inflammatory reaction. However, a previous study demonstrated that patients undergoing endovascular aneurysm repair due to abdominal aortic aneurysm often develop an inflammatory response known as post-implantation syndrome (Sartipy et al. 2015). In these patients, the temperature and WBC count tended to peak on postoperative day 1, while C-reactive protein and procalcitonin tended to peak on postoperative day 3. In our study, the short interval between stenting and onset of inflammation suggests a direct inflammatory reaction to the stent-graft. Additionally, repeated blood culture findings were negative, and there was no fever, no elevation of procalcitonin levels, and no evidence of a bacterial focus elsewhere in the body. Furthermore, no antibiotics were administered and clinical symptoms resolved after steroid therapy. Previously, it was reported that high-grade changes in MRI after implantation were correlated with severity of vessel wall trauma and severity of intimal dissections (Kellner et al. 1997). In the current study, neither IVUS nor post-angioplasty and post-stenting angiography revealed dissection. We therefore excluded vessel wall trauma as the cause of inflammation. Another explanation is an incompatibility reaction to the polytetrafluoroethylene (PTFE) graft. Compared to polyester grafts, PTFE grafts result in a less intense inflammatory response (Sartipy et al. 2015). The Hemobahn stent-graft, which was the previous form of the Viabahn stent-graft, is made using expanded PTFE; however, inflammatory reactions to this device have been reported (Juergens et al. 2002). Additionally, unlike the Hemobahn model, the Viabahn model is heparin coated. Severe perivascular inflammation is observed more often around heparin-coated stent-grafts than around non-coated stent-grafts (Schurmann et al. 1997). Such a response may cause severe clinical symptoms or reduce patency of endovascular prostheses (Link et al. 1996a,b) and needs to be diagnosed and treated immediately.

In the present case, we evaluated the stent-graft via MRI. In a study of aortic grafts, MRI and macroscopic evaluation were compared. Macroscopic evaluation revealed a pronounced thickening of the vascular wall next to the stent-graft, and soft tissue adhesions; some cases formed a tight capsule around the Dacron-covered stent-graft due to perigraft inflammation (Schurmann et al. 1997). In the current case, soft tissue edema was also visible upon MRI. Although we could not perform histopathologic analysis, vascular wall thickening and adhesions around the Viabahn stent-graft were deemed likely upon MRI analysis.

Regarding treatment methods, it is reported that steroid treatment before implantation of a stent-graft can reduce the inflammatory response after endovascular abdominal aortic aneurysm repair, without increasing postoperative infection (Maruta et al. 2016). Steroid treatment might also be effective against a perigraft inflammatory reaction to a Viabahn stent-graft. However, such treatment increases the risk of infection (Stuck et al. 1989). Therefore, we initiated steroid treatment at low dosage for a short period, which immediately lowered inflammation and improved symptoms.

In conclusion, perigraft inflammatory reaction can occur in reaction to a Viabahn stent-graft implant. It can be immediately diagnosed via MRI and treated with steroids to reduce the possibility of occlusion of the stent-graft.

## Data Availability

Not applicable.

## Abbreviations

SFA: Superficial femoral artery
MRI: Magnetic resonance imaging
CT: Computed tomography
EVT: Endovascular treatment
IVUS: Intravascular ultrasound
PTFE: Polytetrafluoroethylene
